# The Adjunctive Role of Hyperbaric Oxygen Therapy in Microbial Infection-Related Conditions

**DOI:** 10.7150/ijms.127803

**Published:** 2026-05-11

**Authors:** Wentian Zhang, Xinxin Li, Hongwei Ma, Ya Li, Yuanhang Xi, Wenlan Wang, Yong Liu, Peijun Han

**Affiliations:** 1 Department of Aerospace Hygiene, School of Aerospace Medicine, Air Force Medical University: Fourth Military Medical University, Xi'an, Shaanxi, China.; 2 School of Medicine, Northwest University, Xi'an, Shaanxi, China.; 3 Department of Microbiology, School of Preclinical Medicine, Air Force Medical University: Fourth Military Medical University, Xi'an, Shaanxi, China.

**Keywords:** Hyperbaric Oxygen Therapy (HBOT), Bacterial Infections, Viral Infections, Antimicrobial Mechanisms, Immunomodulation, Adjunctive Therapy

## Abstract

Hyperbaric oxygen therapy (HBOT) demonstrates expanding applications in infectious diseases through its multimodal mechanisms. As an adjunctive treatment, HBOT directly exerts antimicrobial effects through oxygen toxicity and reactive oxygen species generation, while indirectly enhances host immunity by improving neutrophil function and promoting tissue repair. Clinical evidence supports its adjunctive use in complex infections including diabetic foot wounds, necrotizing soft tissue infections, COVID-19, and mucormycosis, particularly in hypoxic wounds where conventional therapies show limited efficacy. While current studies are promising, further randomized trials are needed to standardize protocols and confirm efficacy.

## Introduction

Hyperbaric Oxygen Therapy (HBOT) has progressed significantly since its conceptual origins in the 17th century with Henshaw's pressurized air chambers^1^. The 19th century brought key scientific foundations, notably Paul Bert's work on oxygen toxicity and the "Paul Bert effect"^2^. Throughout the 20th century, HBOT became standard in diving medicine for decompression sickness and arterial gas embolism, solidifying its role in acute gas exchange disorders. The therapy involves inhalation of 100% oxygen at pressures typically ranging from 2.0 to 3.0 atmospheres absolute (ATA)^3^. These mechanisms drive a range of therapeutic responses, including angiogenesis stimulation, modulation of immune function, and direct antibacterial activity^4^, ^5^.

While HBOT is currently approved by the Undersea and Hyperbaric Medical Society (UHMS) and the 10th European Committee for Hyperbaric Medicine (ECHM) for 14 indications—including carbon monoxide poisoning, diabetic foot ulcers, and radiation-induced tissue injury—growing evidence supports its potential for emerging applications in neurological and inflammatory conditions^6^-^8^. Particularly relevant is its capacity to improve infection control in ischemic or hypoxic wounds, where traditional antibiotics may show limited efficacy due to poor perfusion. Given the rise in antibiotic-resistant infections^9^, adjunctive therapies such as HBOT are gaining renewed interest^10^. This article reviews the established and emerging applications of HBOT in the management of pathogenic bacterial infections, with a focus on the molecular and cellular mechanisms underlying its antimicrobial and host-directed effects.

## Methods

This review provides a comprehensive overview of the adjunctive role of hyperbaric oxygen therapy (HBOT) in microbial infections. A literature search was conducted in the PubMed database using key terms related to HBOT and various infectious disease categories (bacterial, viral, and fungal), with the cutoff date of March 10, 2025. Two reviewers independently screened the titles and abstracts of the retrieved records to select publications most relevant to the scope of this review. The selected full-text articles were then reviewed in depth to synthesize the current understanding of HBOT's mechanisms and its clinical applications. This article elaborates on the fundamental mechanisms of HBOT, then discusses its therapeutic principles and clinical evidence across different types of infectious diseases, concluding with a summary of its therapeutic role in clinical management.

### Mechanisms of HBOT

HBOT exerts its therapeutic effects through multiple interconnected mechanisms, which can be broadly categorized into physical, cellular, and antimicrobial effects^3^. By administering 100% oxygen at pressures above atmospheric level (typically 2.0-3.0 ATA), HBOT fundamentally enhances oxygen delivery to tissues and modulates a range of physiological responses^3^, ^11^.

### Enhancement of Oxygen Delivery

The primary effect of HBOT is a physics-based increase in plasma-dissolved oxygen, which alleviates tissue hypoxia. This principle is effectively applied in clinical emergencies^3^, ^5^. For example, in carbon monoxide poisoning, HBOT competitively displaces CO from hemoglobin and supports cellular respiration via oxygen physically dissolved in plasma^3^, ^12^. Similarly, in decompression sickness, the combined application of Boyle's law (bubble volume reduction) and Henry's law (gas dissolution) under hyperbaric conditions accelerates nitrogen elimination and restores perfusion^3^, ^13^, ^14^.

### Activation of Cellular and Immune Responses

HBOT activates complex signaling pathways that promote repair and modulate inflammation. A key mechanism involves the stabilization of hypoxia-inducible factor-1α (HIF-1α), leading to the upregulation of angiogenic factors such as Vascular Endothelial Growth Factor (VEGF), which is crucial for wound healing in Diabetic Foot Ulcers (DFUs)^4^, ^15^. HBOT also stimulates the activity of endothelial nitric oxide synthase (eNOS) in the bone marrow to mobilize stem/progenitor cells (SPCs) and enhance their functionality in wounds^4^. Moreover, the continuous release of oxygen can promote the survival and migration of keratinocytes and dermal fibroblasts, enhance the expression of angiogenic factors, and reduce the expression of inflammatory cytokines in diabetic wounds^16^. Concurrently, HBOT shifts the immune response to a controlled state by reducing pro-inflammatory cytokines (e.g., IL-6, TNF-α) and promoting anti-inflammatory mediators (e.g., IL-10), as demonstrated in experimental sepsis models^4^, ^17^.

### Antimicrobial Actions

The antimicrobial activity of HBOT operates through complementary direct and indirect pathways^3^, ^13^, ^14^, ^17^. Direct mechanisms involve the generation of reactive oxygen species (ROS)—including superoxide anion (O_2_^-^), hydrogen peroxide (H_2_O_2_), and hydroxyl radicals (•OH)—which disrupt the microbial redox balance and induce lethal oxidative stress^14^, ^17^. This ROS-mediated damage manifests as DNA strand breaks, protein dysfunction, and lipid peroxidation, ultimately causing microbial cell death^18^. This effect is particularly pronounced in anaerobic organisms such as *Clostridium perfringens*, which possess limited antioxidant capacity. Moreover, sustained ROS production under hyperoxia contributes to biofilm disruption and also affects certain aerobic bacteria and fungi^3^, ^17^, ^19^. Indirectly, HBOT augments host innate immunity primarily by enhancing neutrophil function. Under hypoxic conditions, neutrophil respiratory burst activity is compromised; HBOT restores tissue oxygen availability, thereby revitalizing oxidative killing mechanisms and improving phagocytic clearance of pathogens such as *Staphylococcus aureus*^17^. Concurrently, HBOT inhibits neutrophil β2-integrin (Mac-1) activity through a nitric oxide (NO)-mediated process, reducing their adhesion to vascular endothelial cells and facilitating migration to sites of infection, which enhances local antimicrobial efficacy^17^, ^20^-^23^.

### Antibiotic Enhancement

HBOT significantly enhances the efficacy of antibiotics through both direct and indirect mechanisms, positioning it as a valuable adjunctive strategy against drug-resistant bacterial infections and biofilm-associated conditions^24^. For instance, HBOT at 2.8 ATA directly inhibits the growth of *Pseudomonas aeruginosa*, while HBOT at 3.0 ATA delays the logarithmic growth phase of *Staphylococcus aureus*^24^. Additionally, prolonged exposure significantly reduces the minimum inhibitory concentration (MIC) of various antibiotics, thereby exerting synergistic bactericidal effects^24^, ^25^. Indirectly, HBOT contributes to immune modulation and biofilm disruption. In terms of immune enhancement, HBOT increases the partial pressure of oxygen in infected tissues, thereby augmenting the phagocytic capacity and ROS production of neutrophils to support pathogen clearance^26^. Lerche *et al*. demonstrated that HBOT alleviates local tissue hypoxia, improves bacterial elimination in infective endocarditis, and reduces the risk of associated complications^27^. Regarding biofilm disruption, HBOT significantly increases oxygen penetration depth, disrupts the hypoxic microenvironment within biofilms, and enhances antibiotic diffusion and bactericidal activity^26^. Gade *et al*. reported that HBOT nearly quadruples oxygen penetration depth in *P. aeruginosa* biofilms, markedly boosting the efficacy of ciprofloxacin^28^. Similarly, Kolpen *et al*. showed that HBOT restores antibiotic activity against bacteria embedded deep within biofilms^29^. Moreover, HBOT may alter bacterial metabolic states and outer membrane protein expression, thereby modulating antibiotic uptake efficiency^30^, ^31^. Clinically, combining HBOT with antibiotic therapy has been shown to significantly reduce mortality in patients with necrotizing soft tissue infections^32^. Collectively, HBOT enhances antibiotic effectiveness through a multifaceted approach—modulating host immunity, disrupting biofilm architecture, exerting direct bacteriostatic effects, and altering bacterial physiology—offering a promising therapeutic avenue for managing refractory infections.

### The Clinical Application of HBOT in Bacterial Infections

HBOT, which involves the administration of 100% oxygen at pressures exceeding 1 atmosphere absolute, has emerged as a valuable adjunctive treatment in the management of bacterial infections^24^, ^33^-^35^. Its efficacy stems from the dual mechanisms of directly influencing bacterial viability and augmenting host defense responses^4^, ^15^-^17^, ^19^, ^36^. This section provides a comprehensive examination of the therapeutic potential of HBOT, analyzing its effects on bacterial biological characteristics as well as its clinical application across a spectrum of diseases associated with bacterial pathogens.

### Effects of HBOT on Bacterial Biological Characteristics

HBOT significantly influences bacterial behavior through multiple mechanisms, primarily mediated by increased oxygen tension^17^, ^19^, ^36^. Evidence from clinical and experimental studies demonstrates its ability to alter bacterial growth, susceptibility, and microbial community composition^24^, ^34^, ^35^. In a clinical study involving 92 inpatients with *Clostridioides difficile* infection (CDI), Muroya *et al*. reported that the 16 patients who received adjunctive HBOT exhibited a significantly lower recurrence rate and shorter symptom duration compared to the control group, suggesting a beneficial role in managing CDI^34^. At the experimental level, Chmelař *et al*. examined the effects of HBOT on common Gram-negative pathogens, including *Pseudomonas aeruginosa*,* Escherichia coli*, *Klebsiella pneumoniae*, and *Proteus mirabilis*. Under hyperbaric conditions (2.8 ATA), *P. aeruginosa* growth was completely inhibited across varying temperatures and lag phases—a reversible effect upon return to normobaric conditions. Notably, no growth inhibition occurred at 1.9 or 2.4 ATA, indicating a pressure-dependent threshold for antibacterial activity^24^. Beyond direct bacterial suppression, HBOT also modulates microbiota composition. Research by Tee-Tau Eric Nyam *et al*. revealed that HBOT increased the abundance of anaerobic bacteria such as *Prevotella*, which correlated negatively with pro-inflammatory pathways including NOD-like receptor signaling and proteasome activity^35^. This suggests that HBOT may indirectly influence infection outcomes through ecological and immunomodsulatory mechanisms.

### HBOT in Burn-Related Infections

Burn injuries disrupt the skin's structural integrity, creating a hypoxic and nutrient-rich environment that facilitates bacterial colonization and biofilm formation^37^. Localized infections may progress to systemic sepsis, particularly in cases of extensive burns, underscoring the need for effective infection control strategies^37^-^39^. HBOT exerts several antibacterial effects in burn management. By significantly increasing tissue oxygen levels, it enhances leukocyte-mediated phagocytosis and oxidative killing of pathogens. The elevated oxygen tension also potentiates the efficacy of certain antibiotics and inhibits the growth of anaerobic bacteria^17^. Furthermore, HBOT mitigates bacterial proliferation by reducing wound edema and improving perfusion, thereby supporting the delivery of immune cells and antimicrobial agents to the infected site^40^. Clinical studies suggest that adjunctive HBOT can reduce infection-related complications, lower the incidence of sepsis, and decrease the need for surgical interventions in burn patients^41^-^43^. Nawal Khan *et al*. and Ümit Özdemir *et al*., among others, reviewed the articles on the application of HBOT in burn treatment. Most of the studies reported the positive effects of HBOT, including the accelerated healing of burn wounds and the improvement of related complications (such as edema and pain)^43^, ^44^. Similarly, a retrospective literature analysis conducted by Christian Smolle *et al*. indicated that HBOT was associated with diminished edema, improved wound healing, reduced infection or bacterial proliferation, and alleviated pain in the majority of documented cases^41^. Furthermore, in an animal model by Mendy Hatibie Oley *et al*., burns were induced on the shoulder region of 38 rabbits using heated metal plates. Bacterial cultures obtained on days 5 and 10 post-burn revealed a significant reduction in bacterial growth in the HBOT group compared to the control group^42^. However, current evidence remains limited, and further well-designed trials are needed to establish standardized protocols regarding timing, pressure, and duration of HBOT specifically for infection control in burn care^40^, ^45^.

### HBOT in Septic Conditions

In the management of septic conditions, particularly those involving anaerobic and mixed bacterial infections, HBOT offers distinct therapeutic advantages. Sepsis is often characterized by tissue hypoxia, microcirculatory dysfunction, and infection with obligate anaerobes or mixed flora, which may contribute to antibiotic failure and disease progression^46^. HBOT exerts direct bactericidal effects against anaerobic pathogens by generating reactive oxygen species under hyperoxic conditions, thereby inhibiting their growth and toxin production^14^. Preclinical studies have demonstrated that HBOT significantly reduces the number and size of abscesses caused by mixed anaerobic organisms such as *Fusobacterium* and *Bacteroides* species in experimental models, supporting its efficacy as an adjunct to conventional therapy^47^. Furthermore, HBOT enhances the efficacy of antibiotics against biofilm-associated infections commonly encountered in sepsis—such as those caused by *P.aeruginosa*—by restoring aerobic respiration, increasing bacterial metabolic activity, and promoting the generation of lethal oxidative stress within biofilms^29^. These multifaceted actions suggest that incorporating HBOT into the treatment regimen for septic patients with anaerobic or mixed infections may improve bacterial clearance, mitigate infection source, and potentially reduce sepsis-related complications.

### HBOT in Periodontal Disease

Periodontitis is a highly prevalent chronic inflammatory disease worldwide, characterized by the progressive destruction of periodontal supporting tissues^48^, ^49^. The disease is primarily driven by anaerobic bacteria, including *Porphyromonas gingivalis*, *Fusobacterium nucleatum*, and *Tannerella forsythia*, which form pathogenic biofilms and induce persistent inflammatory responses^50^-^52^. HBOT has been shown to be effective as an adjunctive treatment for periodontitis. The mechanism of action involves the direct inhibition of anaerobic bacterial growth through elevated oxygen tension, coupled with enhanced host immune response and tissue repair processes^53^, ^54^. In a randomized study by Alexandru Burcea *et al*. involving 71 patients with periodontitis, those assigned to HBOT showed significantly greater improvements in in oral health indicators—including the Oral Hygiene Index-Simplified (OHI-S), sulcus bleeding index (SBI), tooth mobility (TM), and periodontal pocket depth (PD)^55^. In another randomized trial, Giorgio Lombardo *et al*. divided 20 patients into a test group, which received full-mouth ultrasonic subgingival debridement combined with HBOT, and a control group, which underwent debridement alone. The HBOT group exhibited a significant reduction in probing bleeding. Although both groups demonstrated immediate reductions in bacterial levels post-treatment, the control group experienced faster bacterial recolonization at the three-month follow-up, suggesting a potential role for HBOT in sustaining microbiological improvements^56^. These findings support the use of HBOT as a valuable adjunct to conventional periodontal therapy, especially in populations with compromised healing capacity. However, its broader clinical implementation is constrained by factors such as high equipment costs, extended treatment protocols, and procedural risks.

### HBOT in Osteomyelitis

Osteomyelitis, a severe bone infection, poses significant therapeutic challenges due to its frequent progression to chronicity and the formation of bacterial biofilms that impede antibiotic penetration^57^. *Staphylococcus aureus* is the predominant pathogen involved^58^. The complex pathophysiology of this condition often leads to progressive bone destruction, and in cases such as diabetic foot-related osteomyelitis, it substantially increases the risk of amputation and mortality^59^. HBOT serves as a valuable adjunct to standard surgical and antimicrobial therapy. Its mechanisms of action include significantly increasing oxygen tension in the infected bone, which enhances neutrophil-mediated bacterial killing, synergizes with antibiotics, and promotes osteoblast function and angiogenesis^4^, ^16^. These combined effects help counteract the hypoxic and immune-compromised microenvironment characteristic of chronic osteomyelitis^17^, ^60^-^62^. A study by Menekşe *et al*. involving 80 patients with chronic refractory foot osteomyelitis demonstrated that the combination of surgical debridement, targeted antibiotics, and HBOT (2.5 ATA, 120 min/session, 5 sessions/week for an average of 50 sessions) resulted in a remarkable 85% rate of infection clearance, with outcomes sustained over a 36-month follow-up period^63^. Despite robust evidence of efficacy, the application of HBOT in osteomyelitis is constrained by the extended treatment duration, logistical complexities, and high associated costs, necessitating careful patient selection^64^.

### HBOT in Necrotizing Soft Tissue Infections (NSTIs)

NSTIs are life-threatening conditions characterized by rapid progression and high mortality rates^65^. Common pathogens include Group A *Streptococcus* and *Escherichia coli*^66^. The aggressive nature of these infections necessitates prompt diagnosis and comprehensive treatment to mitigate systemic complications and reduce amputation risk^67^. As an adjunct to urgent surgical debridement and broad-spectrum antibiotic therapy, HBOT exerts beneficial effects through multiple pathways. It enhances tissue oxygen delivery, thereby supporting neutrophil-mediated bacterial clearance and inhibiting anaerobic bacterial growth. Additionally, HBOT helps modulate the excessive inflammatory response and reduces tissue edema, contributing to the preservation of viable tissue^32^, ^68^, ^69^. In addition, clinical observations indicate that the integration of HBOT into NSTI management may lead to reduced mortality, lower amputation rates, decreased frequency of surgical debridements, and shorter ICU and hospital stays^32^, ^69^, ^70^. The mechanism of HBOT for NSTIs is related to oxidative stress and the regulation of inflammatory pathways. Morten Hedetoft *et al*. investigated the impact of HBOT on oxidative stress markers in patients with NSTI. The results indicated that HBOT was associated with significant increases in myeloperoxidase and superoxide dismutase (SOD) levels. Moreover, this oxidative stress response was more pronounced in patients presenting with septic shock^71^. In a related study, Julie Vinkel *et al*. performed whole-transcriptome RNA sequencing on blood samples from 85 intensive care unit-admitted NSTI patients, collected before and after HBOT. Transcriptomic analysis revealed that HBOT modulated key genes involved in helper T cell activation and downregulated highly inflammatory pathways, including NF-κB, which are typically upregulated in NSTI^72^. However, the current evidence is predominantly derived from retrospective studies, and the timing of HBOT administration requires careful consideration. Prospective, randomized controlled trials are needed to more definitively establish the efficacy, safety, and optimal implementation protocol of HBOT as an adjunctive therapy for NSTIs.

### HBOT in Diabetic Foot Infections

Diabetic foot ulcers (DFUs) represent a severe and frequent complication of diabetes, often leading to substantial morbidity and impaired quality of life^73^, ^74^. These wounds are commonly complicated by polymicrobial infections, which occur in the setting of neuropathy, peripheral artery disease, and impaired healing—factors that significantly increase the risk of lower extremity amputation and mortality^75^, ^76^. HBOT has been increasingly incorporated into the multidisciplinary management of DFUs^77^, ^78^. Its therapeutic benefits are attributed to the elevation of tissue oxygen levels, which enhances leukocyte bactericidal activity, supports antibiotic penetration, and promotes angiogenesis and collagen synthesis—all of which are critical in controlling infection and facilitating wound repair^4^, ^5^. Multiple meta-analyses have consistently report that adjunctive HBOT significantly improves DFU healing rates and reduces the incidence of major amputations^79^-^81^. In a related study, Erdinç Ercan *et al*. assessed the impact of HBOT on hematological in patients with diabetic foot. Following treatment, lymphocyte and eosinophil counts, mean corpuscular hemoglobin concentration, and red cell distribution width increased^82^. The increase in immune cells indicates to some extent that the body's antibacterial ability has improved. Despite these encouraging findings, further well-designed trials are needed to refine treatment protocols—such as optimal pressure, session frequency, and patient selection criteria—and to confirm the long-term benefits of HBOT in diabetic foot infection outcomes.

### HBOT in Necrotizing fasciitis (NF)

Necrotizing fasciitis (NF) is a life-threatening soft tissue infection typically arising from odontogenic, pharyngeal, or cutaneous sources and involves pathogens such as Group A *Streptococcus*, *Staphylococcus* aureus, and mixed aerobic/anaerobic bacteria^68^, ^83^. It is characterized by rapid fascial necrosis and systemic toxicity, and currently relies on radical surgical resection, broad-spectrum antibiotics, and adjuvant Hyperbaric medicine^83^-^85^. Clinical experience supporting the use of HBOT is primarily based on case series and retrospective studies, with substantial variability noted in both treatment protocols and reported outcomes. Thrane *et al*. administered HBOT at 2.8 ATA for 90 minutes per session to 30 patients with head and neck necrotizing fasciitis (NF-HN)^84^. Given that HBOT did not reduce mortality in patients with NF and may increase the risk of complications and sequelae, its use might be best reserved as a selective adjunctive intervention in specific NF patient subgroups. In contrast, Langford *et al*. reported 100% survival in six patients with cervical NF treated with HBOT (2.0-3.06 ATA, 60-120 minutes per session), markedly surpassing the historical mortality rate of 28%^86^. In a 2024 review, Kryeziu *et al*. summarized 16 studies on HBOT combined with repeated surgical debridement and antibiotics for NF^87^. The analysis indicated that HBOT may reduce mortality—for example, from 66% to 23%—and could serve as a beneficial adjunct, particularly in anaerobic infections. Mladenov *et al*. conducted a 10-year analysis comparing outcomes in 192 patients with NF/Fournier's gangrene^88^. They reported comparable survival rates between patients receiving HBOT (2.96 ATA, 90 min) and non-HBOT patients (73.5% vs 75.5%). Notably, despite the HBOT group having more severe disease—including a higher sepsis prevalence (61.4%)—their similar outcomes suggest that adjunctive HBOT may aid in controlling severe complications and promoting wound healing^88^. Thus, HBOT serves only as a conditional adjunct in clinical practice, indicated for stabilized cases with extensive necrosis or anaerobic infection but avoided in hemodynamically unstable patients due to transport-related risks. Future efforts should prioritize multicenter randomized controlled trials to clarify its efficacy in specific NF subtypes and to establish clear criteria for patient selection^68^, ^83^, ^85^-^89^.

### HBOT in Intracranial Abscess (ICA)

Intracranial abscess (ICA) comprises cerebral abscess, subdural empyema, and epidural empyema, sharing similar etiologies primarily through direct invasion or hematogenous spread. Bacterial ICA mainly originates from contiguous Ear, Nose, and Throat (ENT) infections or hematogenous dissemination^90^-^93^. Current management includes medical monitoring, antibiotics, and surgery, with HBOT investigated as an adjunctive therapy in selected cases. The clinical application of HBOT dates to 1955 when Churchill-Davidson and colleagues first explored its potential to enhance radiotherapy effects in cancer patients^90^. In a more recent cohort study by Bartek Jr *et al*. involving 20 spontaneous brain abscess patients receiving adjuvant HBOT, only 14% experienced recurrence and 80% achieved favorable outcomes (Glasgow Outcome Scale score of 5), surpassing results in the non-HBOT group receiving standard care alone^93^. Given limited evidence, future multicenter RCTs are needed to validate HBOT efficacy across ICA subtypes and patient subgroups. Concurrent efforts should identify predictive biomarkers for patient selection and conduct cost-effectiveness analyses to evaluate long-term clinical benefits^90^-^93^.

### HBOT in Single bacteria

Current research on HBOT for single bacterial species remains limited, with existing studies primarily focusing on common pathogens such as *Staphylococcus aureus* and *P. aeruginosa*^25^, ^28^, ^94^-^99^. In cystic fibrosis (CF) patients, chronic pulmonary infections caused by *P. aeruginosa* represent a major complication, characterized by the formation of antibiotic-resistant biofilms or aggregates in bronchial mucus^94^. As an adjunctive strategy, HBOT has consistently demonstrated *in vitro* efficacy in enhancing the bactericidal effects of antibiotics against *P. aeruginosa*^95^-^98^.

Early research on the physiological effects of HBOT on *P. aeruginosa* laid the groundwork for understanding its targeted antibacterial properties. In 2016, Kolpen *et al*. utilized an agarose-embedded PAO1 biofilm model mimicking the hypoxic sputum environment in CF and found that HBOT (2.8 ATA) significantly increased the bactericidal activity of ciprofloxacin^95^. This was further elucidated in a 2018 reaction-diffusion modeling study by Gade *et al*., which demonstrated that HBOT improves oxygen penetration into deeper biofilm regions, thereby eradicating hypoxic bacterial subpopulations^28^. Translating these findings to clinical strains, Møller *et al*. observed in an ex vivo aggregate model derived from CF patients that HBOT (2.8 ATA, 90 minutes) enhanced the efficacy of tobramycin across all tested isolates^97^. Collectively, these *in vitro* studies support HBOT as a promising adjunct therapy for *P. aeruginosa* infections in CF. However, current evidence remains confined to laboratory models. Future efforts should prioritize *in vivo* studies using animal models of chronic pulmonary infection to optimize HBOT protocols, assess potential risks such as airway obstruction, and evaluate long-term safety in patients with contraindications.

*Staphylococcus* (*S. aureus*) is a common Gram-positive pathogen responsible for a wide range of infections. Its capacity to form biofilms and adapt to hypoxic conditions diminishes susceptibility to antibiotics and host immune defenses^99^. As an adjunctive strategy, HBOT has been shown to enhance antibiotic efficacy against *S. aureus* and modulate host inflammatory responses, findings consistently supported by both *in vitro* and *in vivo* studies^25^, ^96^. Early evidence dates back to Bornside's pioneering work in 1967, which employed a static broth culture model to assess the effect of HBOT on *S. aureus* ATCC 6538P^25^. The study demonstrated a 60% reduction in logarithmic-phase bacterial growth under hyperbaric conditions compared to normoxia, along with a progressive decrease in the MIC of multiple antibiotics, including penicillin and streptomycin. These *in vitro* findings have been corroborated by recent *in vivo* investigations. For instance, Lerche *et al*. established a rat model of *S. aureus* infective endocarditis that closely mimics human disease, confirming that HBOT enhances antibacterial activity, reduces tissue bacterial load, and attenuates inflammatory responses^96^. Nonetheless, several limitations remain. Future research should focus on optimizing HBOT parameters, evaluating its synergy with other antimicrobial agents and conducting multicenter randomized controlled trials to validate its clinical efficacy and safety in humans.

### The Clinical Application of HBOT in Viral Infections

Building upon its established role in bacterial infections, HBOT is increasingly investigated for its potential in managing viral diseases. The therapeutic rationale extends beyond improved oxygenation to encompass immunomodulatory and direct antiviral effects.

### HBOT in SARS-CoV-2 (COVID-19)

COVID-19, caused by the SARS-CoV-2 virus, initiates infection via binding to ACE2 receptors, resulting in multi-system damage^100^, ^101^. HBOT addresses these mechanisms by enhancing oxygen delivery, attenuating inflammation (e.g., reducing IL-6, TNF-α), and improving microcirculation^102^. Clinical studies have supported the efficacy and safety of HBOT^3^, ^100^, ^103^-^111^. A systematic review involving 37 hypoxemic COVID-19 patients reported that among 26 evaluated participants, 24 avoided intubation and 23 survived, with no serious adverse events, suggesting HBOT as a promising intervention pending further validation by randomized controlled trials^103^. In a randomized, sham-controlled, double-blind trial including 73 patients with persistent symptoms at least 3 months after COVID-19, the HBOT group (n = 37, 40 sessions) demonstrated significant improvements in cognition, energy, and sleep, along with optimized cerebral perfusion and microstructure, compared to the sham group (n = 36)^111^. Another multicenter randomized controlled trial indicated that among patients with long COVID, HBOT (1.47 ATA) significantly increased hypoxemia remission rates and shortened the median recovery time compared to standard care (3 vs. 9 days)^112^. By targeting SARS-CoV-2, HBOT-generated reactive oxygen species inhibit viral envelope assembly and reduce viral replication. It also promotes the clearance of persistent virus by ameliorating the host microenvironment, contributing to the management of COVID-19^113^. In a Swedish exploratory randomized controlled trial involving 17 critically ill COVID-19 patients with moderate ARDS, adjunctive HBOT demonstrated multi-faceted benefits. At the transcriptomic level, HBOT induced significant changes in peripheral blood mononuclear cells (PBMCs), characterized by 791 differentially expressed genes and an endoplasmic reticulum (ER) stress signature. Clinically, HBOT was associated with shorter hospital stays, reduced National Early Warning Scores (NEWS), and improved PaO₂/FiO₂ ratios^114^. Mechanistically, HBOT appears to modulate COVID-19 immunopathology through dual pathways: by suppressing pro-inflammatory factor expression and inflammatory cell infiltration to prevent cytokine storm, while concurrently restoring immune cell function through enhanced lymphocyte and natural killer cell activity^114^, ^115^. HBOT shows potential in improving quality of life, reducing fatigue and cognitive impairment, alleviating neuropsychiatric symptoms, and enhancing cardiopulmonary function^109^, ^111^, ^114^. However, further large-scale trials are needed to optimize its treatment protocols.

### Other Viral Infections

Beyond COVID-19, HBOT has been explored as an adjunct therapy for several other viral conditions, though the evidence base is less established^27^, ^96^, ^116^-^122^. In the context of allogeneic hematopoietic stem cell transplantation, HBOT (1.4-2.0 ATA) has shown promise in managing BK polyomavirus-associated hemorrhagic cystitis (BKV-HC), with observational studies reporting complete clinical remission in 86% of patients and reductions in viral load, likely mediated by enhanced tissue oxygenation and mucosal healing^118^, ^121^. Preliminary *in vitro* research suggests that HBOT may inhibit HIV-1 replication, while small clinical series indicate potential for alleviating antiretroviral-related neuropathy, improving quality-of-life scores without significantly altering CD4+ counts^116^, ^122^. Similarly, *in vitro* models of HPV-associated lesions suggest HBOT can exert anti-proliferative effects^123^. Animal studies indicate that HBOT may modulate host responses and mitigate pathological processes in infective endocarditis^27^. HBOT presents a multifaceted therapeutic approach to viral infections, primarily through modulating host responses—improving oxygenation, reducing inflammation, and promoting tissue repair^3^, ^108^, ^124^. While clinical results for COVID-19 are encouraging, evidence for other viral diseases is nascent and requires confirmation through larger, well-designed randomized controlled trials. Future research should focus on elucidating definitive antiviral mechanisms, standardizing treatment protocols, and evaluating cost-effectiveness to better define HBOT's role in viral management.

### The Clinical Application of HBOT in Fungal Infections

In contrast to its more established role in bacterial and viral infections, the application of HBOT in fungal diseases has been less extensively studied. However, emerging evidence supports its potential as an adjunctive treatment for invasive fungal infections, such as mucormycosis and aspergillosis, which predominantly affect immunocompromised individuals and are associated with high mortality rates^139^. Clinical experience with HBOT in fungal infections is largely derived from retrospective studies and case series. A study involving 14 patients with invasive fungal infections (including at least 9 cases of mucormycosis) reported a 50% survival rate following adjunctive HBOT, exceeding historical controls^79^. In rhinocerebral mucormycosis, HBOT has been associated with reduced tissue necrosis and improved neurological outcomes. Treatment protocols generally involve 100% oxygen at 2.0-2.5 ATA for 60-90 minutes per session, and are delivered 5-7 times per week, with duration tailored to clinical response^138^. However, its application is constrained by equipment requirements, limited availability, and insufficient data regarding its efficacy in other fungal infections such as aspergillosis. Further well-designed studies are needed to define the optimal role of HBOT within the multidisciplinary management of invasive fungal diseases.

## Conclusion

HBOT demonstrates significant potential in the management of diverse infectious diseases, spanning bacterial, viral, and fungal pathogens. Its efficacy derives from a multi-mechanistic action that combines direct pathogen inhibition—through oxygen toxicity and reactive oxygen species generation—with critical host-directed effects, including enhancement of immune function, modulation of inflammation, and promotion of tissue repair and angiogenesis. Clinical evidence supports its role as a valuable adjunct to conventional antimicrobial therapies, particularly in complex scenarios like diabetic foot infections and mucormycosis. However, broader application is currently constrained by a need for more robust randomized controlled trials to standardize protocols and definitively establish efficacy across various infections. Future research should focus on elucidating detailed molecular mechanisms and optimizing HBOT's integration into antimicrobial stewardship programs.

## Figures and Tables

**Figure 1:**
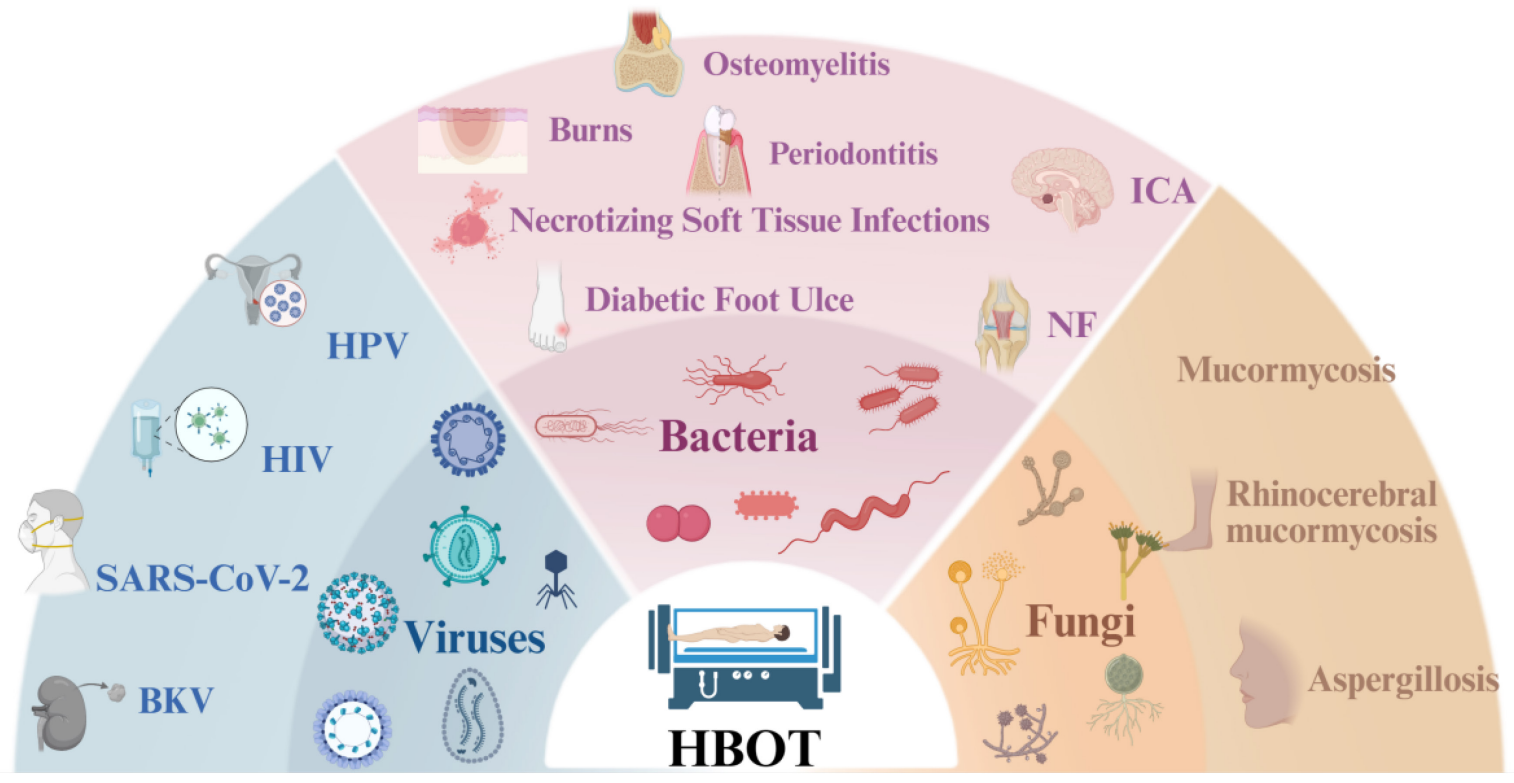
** Application of HBOT in clinical microbial infections.** This schematic summarizes representative protocols for the management of viral, bacterial, and fungal infections.

**Table 1:** Clinical Applications of HBOT in pathogenic microorganisms and infectious diseases

Type	Disease	References
Bacteriological	Burns Periodontitis Osteomyelitis Necrotizing Soft Tissue Infections Diabetic Foot Ulcers Necrotizing Fasciitis (NF) Intracranial Abscess (ICA) Single Bacteria Gas Gangrene Lower Extremity Necrotizing Soft Tissue Infection Toxic Shock Syndrome Malignant otitis externa (MOE) Tuberculosis disease (TB) Spongiosus-cutaneous Fistula Complex Regional Pain Syndrome (CRPS) Toxic Epidermal Necrolysis (TEN) Cystic Fibrosis (CF) Hansen's disease Fournier's Gangrene (FG)	17, 37-45 48-55, 125 4, 16, 17, 57-64 32, 65-72 4, 5, 73-76, 79-82 68, 83, 85-89 90-93 25, 28, 94-99 126 127 128 129 130 131 132 133 97 134 135
Viral	COVID-19 BK polyomavirus (BKV) Human Immunodeficiency virus (HIV) Human Papillomavirus (HPV) Focal Suppurative Infections (FSIs) Hepatitis B Virus (HBV)	3, 100, 103-111 118, 121 116, 122 123 136 137
Fungal	Mucormycosis Aspergillosis Zygomycosis	79, 138 139 140

## Abbreviations

HBOT: hyperbaric oxygen therapy
ATA: atmospheres absolute
UHMS: undersea and hyperbaric medical society
ECHM: european committee for hyperbaric medicine
HIF-1α: hypoxia-inducible factor-1α
VEGF: vascular endothelial growth factor
DFUs: diabetic foot ulcers
eNOS: endothelial nitric oxide synthase
SPCs: stem/progenitor cells
MIC: minimum inhibitory concentration
ROS: reactive oxygen species
CDI: *clostridioides difficile* infection
OHI-S: oral hygiene index-simplified
SBI: sulcus bleeding index
TM: tooth mobility
PD: periodontal pocket depth
NSTIs: necrotizing soft tissue infections
SOD: superoxide dismutase
NF: necrotizing fasciitis
NF-HN: neck necrotizing fasciitis
ICA: Intracranial abscess
ENT: ear, nose, and throat
CF: cystic fibrosis
*S. aureus*: *Staphylococcus*
PBMCs: blood mononuclear cells
ER: endoplasmic reticulum
NEWS: national early warning scores
BKV-HC: BK polyomavirus-associated hemorrhagic cystitis
MOE: malignant otitis externa
TB: tuberculosis disease
CRPS: complex regional pain syndrome
TEN: toxic epidermal necrolysis
FG: fournier's gangrene
BKV: BK polyomavirus
HIV: human immunodeficiency virus
HPV: human papillomavirus
FSIs: focal suppurative infections
HBV: hepatitis B virus
